# Supplementary material for: Deficiency of thyroid hormone receptor protects retinal pigment epithelium and photoreceptors from cell death in a mouse model of age-related macular degeneration
Source: Cell Death Dis. 2022 Mar 21;13(3):255. doi: 10.1038/s41419-022-04691-2 (PMC8938501; doi:10.1038/s41419-022-04691-2)
Supplement: Supplementary file 1 — Supplementary Information [file 41419_2022_4691_MOESM1_ESM.pdf]

For consideration for publication in Cell Death & Disease

Deficiency of thyroid hormone receptor protects retinal pigment epithelium and photoreceptors from cell death in a mouse model of age-related macular degeneration

Hongwei Ma, Fan Yang, and Xi-Qin Ding

Department of Cell Biology, University of Oklahoma Health Sciences Center, Oklahoma City, Oklahoma, USA

Supplementary Information

(Supplementary Figures 1-4, Supplementary Table 1)

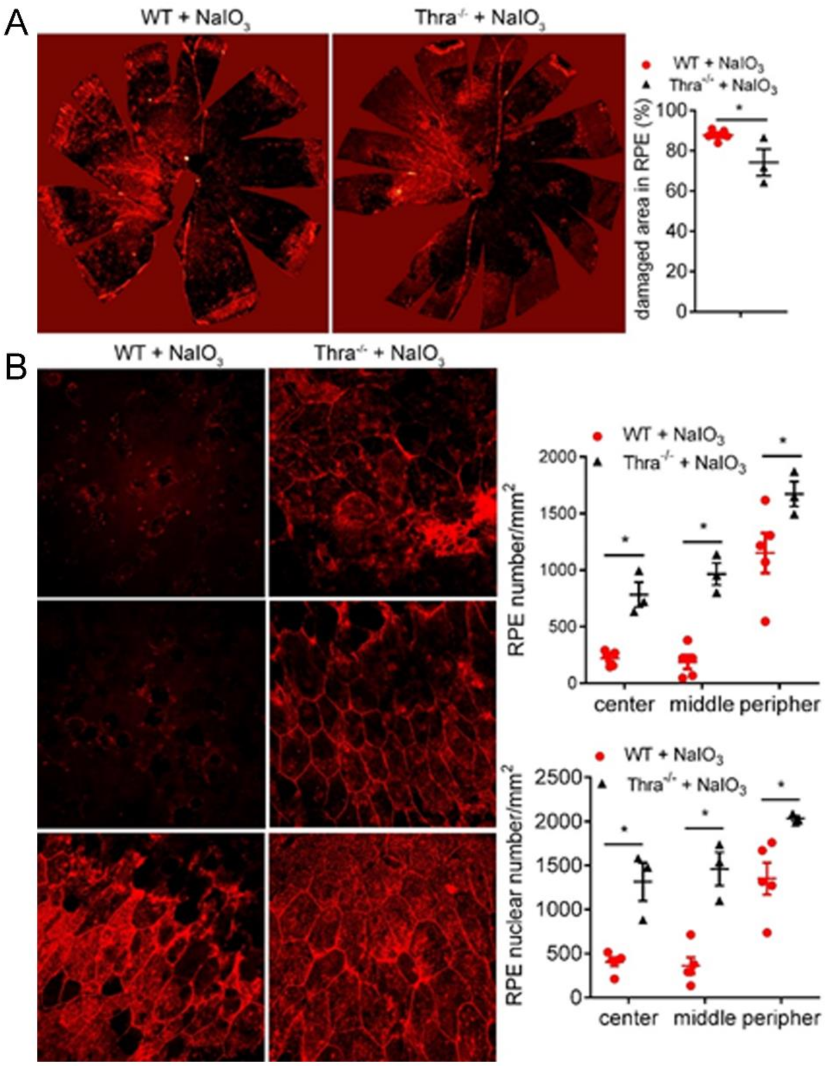

Supplementary Figure 1. Deletion of *Thrα1* protected RPE from damage/cell loss induced by $NaIO_3$ in mice at 7 months. RPE morphology and cell loss were evaluated by phalloidin staining for F-actin and DAPI staining for nucleus on RPE whole mounts prepared from 7 months *Thrα1*$^{-/-}$ and wild-type mice at 2 days post-$NaIO_3$ injection. **A.** Shown are representative low magnification images of phalloidin staining of the damaged area in the RPE and corresponding quantitative analysis of the damaged area. **B.** Shown are representative high magnification images of phalloidin staining taken at different regions of the RPE and corresponding quantitative analysis of RPE cell

numbers. Data are represented the mean ± *SEM* for 3-5 mice per group (* $p < 0.05$; compared with wild-type mice treated with $NaIO_3$).

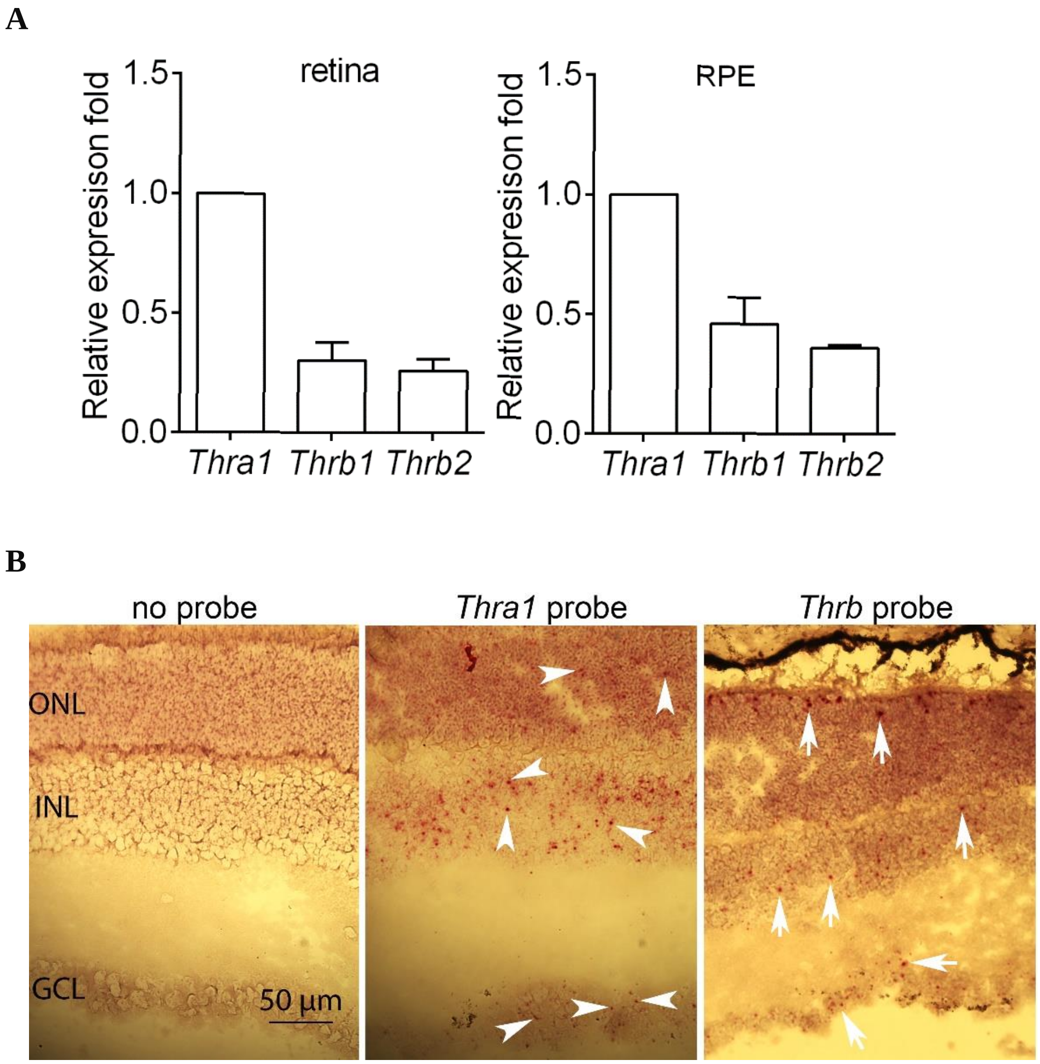

Supplementary Figure 2. Expression of THR subtypes in the retina and RPE. A. qRT-PCR detection of mRNA expression levels of THR subtypes in the RPE and retinas of P15 C57BL/6J mice. Data are represented as means ± *SEM* for 2 assays using RPE and retinas prepared from 3 mice per group. **B.** RNAscope *in situ* hybridization detection of mRNA expression of THR subtypes on the retinal sections prepared from P20 C57BL/6J mice. Shown are representative

images of *Thra1* and *Thrb* labeling on the retinal cross sections. ONL, outer nuclear layer; INL, inner nuclear layer; GCL, ganglion cell layer, arrowheads and arrows denote positive staining of *Thra1* probe and *Thrb* probe, respectively.

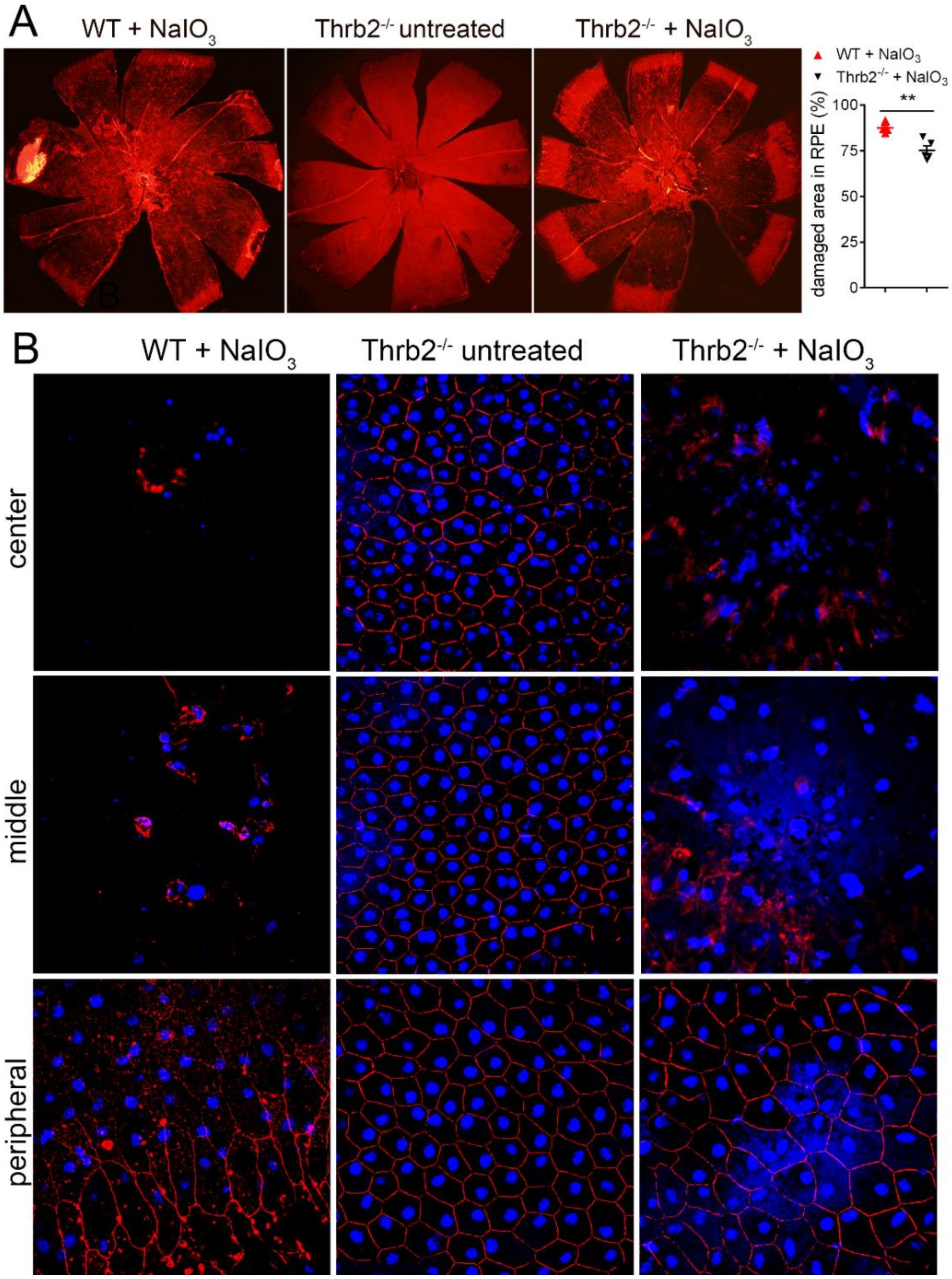

Supplementary Figure 3. Deletion of *Thrb2* protected RPE from damage/cell loss induced by $NaIO_3$ in mice at 17 months. RPE morphology and cell loss were evaluated by phalloidin staining for F-actin and DAPI staining for nucleus on RPE whole mounts prepared from 17 months *Thrb2*$^{-/-}$ and wild-type mice at 2 days post-$NaIO_3$ injection. **A.** Shown are representative low magnification images of phalloidin staining of the damaged area in the RPE and corresponding quantitative analysis of the damaged area. **B.** Shown are representative high magnification images of phalloidin staining and DAPI labeling taken at different regions of the RPE. Data are represented as means ± *SEM* for 3-6 mice per group (** $p < 0.01$; compared with wild-type mice treated with $NaIO_3$).

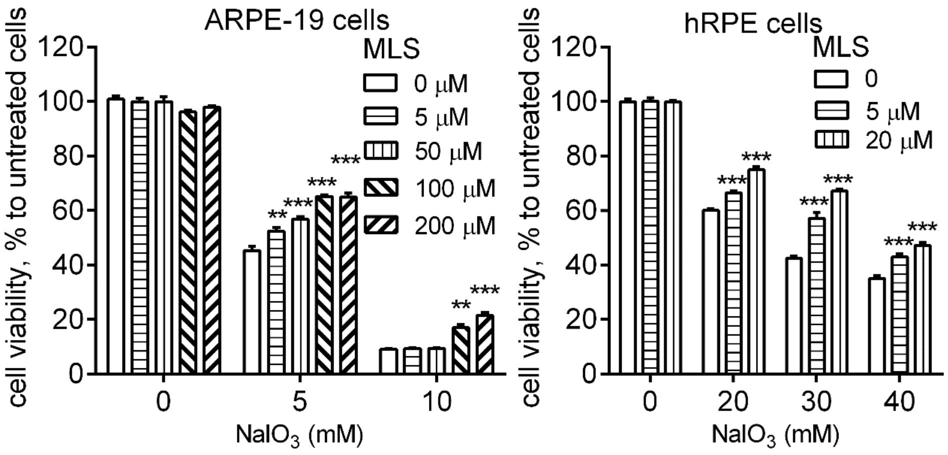

Supplementary Figure 4. Treatment with THR antagonist MLS protected ARPE-19 cells and hRPE cells from $NaIO_3$-induced cell death. ARPE-19 cells and hRPE cells cultured in RPMI-1640 medium were treated with $NaIO_3$ at concentrations indicated in the absence and presence of various concentrations of MLS for 24 hours, and were then analyzed for cell viability by MTS assay. Shown are results of MTS assay in ARPE-19 cells and hRPE cells. Data are

represented as means ± *SEM* for 3 independent experiments. (** $p < 0.01$, and *** $p < 0.001$, compared with cells without MLS treatment).

Supplementary Table 1. Primers used for qRT-PCR

Gene	Forward primer	Reverse primer
Thra1	AGAAGAGTCAGGAGGCCTACCT	CCTACTCCTCATTCCTCCTGA
Thra2	AGAAGAGTCAGGAGGCCTACCT	TGAAGAACCGGCCCTCGGAGACTT
Thrb1	GTTTTCCCTCTCGTCCATCAGAGGACCTG	GCTTCCGCTTGGCTAGCCTCTTGCT
Thrb2	AGTCAGTCCAGCCAGCCTGCACAT	GCTTCCGCTTGGCTAGCCTCTTGCT
Hprt1	GCAAACTTTGCTTTCCCTGGTT	CAAGGGCATATCCAACAACA
Casp3	GACTGATGAGGAGATGGCTTG	TGCAAAGGGACTGGATGAAC
Casp7	CCCACTTATCTGTACCGCATG	GGTTTTGGAAGCACTTGAAGAG
Casp8	AACTTCCTAGACTGCAACCG	TCTCAATTCCAACTCGCTCAC
Gpx4	GCAATGAGGCAAAACTGACG	CTTGATTACTTCCTGGCTCCTG
Nox4	TCCAAGCTCATTTCCCACAG	CGGAGTTCCATTACATCAGAGG
Ucp2	GCATTGGCCTCTACGACTC	AAGCGGACCTTTACCACATC
Gss	GATCCTGTCCAATAACCCCAG	GCACGCTGGTCAAATATGTTC
Ctsb	AGACCTGCTTACTTGCTGTG	GGAGGGATGGTGTATGGTAAG
Ncf1	TCATCCTTCAGACCTATCGGG	ACCTCGCTTTGTCTTCATCTG
Ehd2	AGCTCAACGACCTAGTGAAAC	TCGCAAAGATGACAGGCAG
Ripk1	GGAAGGATAATCGTGGAGGC	AAGGAAGCCACACCAAGATC
Ripk3	TCTTTACTGAGACTCCCGGT	AGTTCCCAATCTGCACTTCAG
Mlkl	ACTGTGAACTTGGAACCCTG	TGCTGATGTTTCTGTGGAGTG
Tradd	ACGAACTCACTAGTCTAGCAGAG	AATACCCCAACAGCCACC
Tnf1α	CTTCTGTCTACTGAACTTCGGG	CAGGCTTGTCACTCGAATTTTG
Tnfrsf1a	CTCTGCTCTACGAATCACTCTG	CACAGCATACAGAATCGCAAG
Tnfrsf9	CCTGTGATAACTGTCAGCCTG	TCTTGAACCTGAAATAGCCTGC
Nlrp3	CTCCAACCATTCTCTGACCAG	ACAGATTGAAGTAAGGCCGG
Il1α	TGCAGTCCATAACCCATGATC	ACAAACTTCTGCCTGACGAG
Il1β	ACGGACCCCAAAAGATGAAG	TTCTCCACAGCCACAATGAG
Il6	CAAAGCCAGAGTCCTTCAGAG	GTCCTTAGCCACTCCTTCTG
Il22	AGCTTGAGGTGTCCAACTTC	GGTAGCACTGATCTTTAGCACTG
