# Supplementary material for: Deficiency of thyroid hormone receptor protects retinal pigment epithelium and photoreceptors from cell death in a mouse model of age-related macular degeneration
Source: Cell Death Dis. 2022 Mar 21;13(3):255. doi: 10.1038/s41419-022-04691-2 (PMC8938501; doi:10.1038/s41419-022-04691-2)
Supplement: Supplementary file 2 — CDDIS-21-3274RR_Ma et al [file 41419_2022_4691_MOESM2_ESM.docx]

*For consideration for publication in Cell Death & Disease*

**Deficiency of thyroid hormone receptor protects retinal pigment epithelium and photoreceptors from cell death in a mouse model of age-related macular degeneration**

Hongwei Ma, Fan Yang, and Xi-Qin Ding

Department of Cell Biology, University of Oklahoma Health Sciences Center, Oklahoma City, Oklahoma, USA

*To whom correspondence should be addressed: Xi-Qin Ding, Department of Cell Biology, University of Oklahoma Health Sciences Center, 940 Stanton L. Young Blvd., BMSB 553, Oklahoma City, Oklahoma 73104, USA; Phone: 1 (405) 271-8001 ext. 47966; Fax: 1 (405) 271-3548; E-mail: xi-qin-ding@ouhsc.edu

**Running title:** Thyroid hormone receptor and RPE/retinal cell death

**Abstract**

Age-related macular degeneration (AMD) is the leading cause of vision loss in the elderly. Progressive dystrophy of the retinal pigment epithelium (RPE) and photoreceptors is the characteristic of dry AMD, and oxidative stress/damage plays a central role in the pathogenic lesion of the disease. Thyroid hormone (TH) regulates cell growth, differentiation and metabolism, and regulates development/function of photoreceptors and RPE in the retina. Population/patients-based studies suggest an association of high free serum TH levels with increased risk of AMD. We recently showed that suppressing TH signaling by anti-thyroid treatment reduces cell damage/death of the RPE and photoreceptors in an oxidative stress/sodium iodate (NaIO_3_)-induced mouse model of AMD. This work investigated the effects of TH receptor (THR) deficiency on cell damage/death of the RPE and photoreceptors and the contribution of the receptor subtypes. Treatment with NaIO_3_ induced RPE and photoreceptor cell death/necroptosis, destruction, and oxidative damage. The phenotypes were significantly diminished in *Thrα1^-/-^*, *Thrb^-/-^*, and *Thrb2^-/-^* mice, compared with that in the wild-type (C57BL/6J) mice. The involvement of the receptor subtypes varies in the RPE and retina. Deletion of *Thrα1* or *Thrb* protected RPE, rods, and cones, whereas deletion of *Thrb2* protected RPE and cones but not rods. Gene expression analysis showed that deletion of *Thrα1* or *Thrb* abolished/suppressed the NaIO_3_-induced upregulation of the genes involved in cellular oxidative stress responses, necroptosis/apoptosis signaling, and inflammatory responses. In addition, THR antagonist effectively protected ARPE-19 cells and hRPE cells from NaIO_3_-induced cell death. This work demonstrates the involvement of THR signaling in cell damage/death of the RPE and photoreceptors after oxidative stress challenge and the receptor subtype contribution. Findings from this work support a role of THR signaling in the pathogenesis of AMD and the strategy of suppressing THR signaling locally in the retina for protection of the RPE/retina in dry AMD.

**Introduction**

Age-related macular degeneration (AMD) is the leading cause of blindness in the elderly ^1, 2, 3^. The dry AMD, also known as geographic atrophy, is a form of slowly progressing geographic atrophy of the macula and comprises a majority of AMD cases (~90%) ^1, 4^. The disease is characterized by a progressive macular degeneration of the retinal pigment epithelium (RPE) and photoreceptors. There are multiple pathological factors, including aging, oxidative stress, chronic inflammation, and genetic defects. However, oxidative stress/damage to the RPE and the subsequent deterioration of photoreceptors is recognized as the core pathogenic lesion of AMD ^1, 5, 6^.

Thyroid hormone (TH) signaling regulates cell growth, differentiation, and metabolic homeostasis ^7, 8, 9^. In the eye, TH signaling regulates retinal/cone development and cone opsin expression ^10,^ ^11, 12, 13, 14^. TH signaling has also been linked to cone viability/cone degeneration. Stimulating TH signaling induces cone death ^11,^ ^12^, whereas suppressing TH signaling improves cone survival in mouse models of Leber's congenital amaurosis (LCA) and achromatopsia ^12, 13, 15^ ^16^. Of note, TH signaling has been implicated in the pathogenesis of AMD. The population/patient-based studies showed that higher free serum TH values were associated with increased risk of AMD ^17, 18, 19,^ ^20, 21^. TH signaling has also been linked to other types of neurodegenerative conditions, including Alzheimer’s disease ^22, 23^.

Using a sodium iodate (NaIO_3_)-induced mouse model of AMD ^24, 25, 26^, we recently showed that treatment with anti-thyroid drug protected RPE and photoreceptors from oxidative damage and cell death/necroptosis and preserved retinal function ^27^. The present work expanded this research by investigating the involvement of TH receptors (THRs) and the contribution of the receptor subtypes. T3 acts through THRs that belong to the nuclear hormone receptor superfamily and function as ligand-dependent transcription factors ^9^. Two genes, *THRA* and *THRB*, encode related receptors across vertebrate species ^9, 28^. THRA1 is encoded by the *THRA* gene, and two THRB isoform splice variants, THRB1 and THRB2, are encoded by the *THRB* gene. These receptor subtypes are broadly expressed in a variety of tissues, including the RPE and retina ^29, 30, 31^. However, THRB2 is expressed only in cones in the retina ^29, 32, 33^. THRB2 has been shown to mediate the regulation of TH in cone opsin expression ^10, 14, 34^ and cone viability ^11, 16^. We examined the NaIO_3_-induced cell damage/death of the RPE and photoreceptors in *Thrα1^-/-^*, *Thrb^-/-^* (resulting in deletion of both *Thrb1* and *Thrb2*), *Thrb2^-/-^*, and wild-type (C57BL/6J) mice. Our results show that deficiency of THR significantly diminished the NaIO_3_-induced cell damage/death and upregulation of the genes involved in cellular oxidative stress responses, activation of the cell death signaling, and inflammatory responses.

**Results**

***Deletion of Thrα1*** ***protected RPE and photoreceptors from damage/cell loss induced by NaIO_3_***

In the previous study, we showed that anti-thyroid treatment reduces NaIO_3_-induced oxidative damage/cell death of the RPE and photoreceptors^27^. The present work investigated the THR mechanisms involved in NaIO_3_-induced damage. We first examined the contribution of THRA1. *Thra1^-/-^*, *Thra1^+/-^*, and wild-type (C57BL/6J) mice received a single injection of NaIO_3_ (30 mg/kg, *i.p.*) at postnatal day 30 (P30), and were then analyzed for RPE morphology and photoreceptor integrity at 3 days post-NaIO_3_ injection. A single administration of NaIO_3_ (*i.v.*, *i.p*. or intraocular injection) induces RPE/photoreceptor damage in experimental animals in a concentration-dependent and time-dependent manner ^25, 35, 36, 37^. The functional and morphological impairments are observed as early as 24 hours, become more severe at 3-7 days, and last up to 4 weeks. We chose 3 days after the treatment as the evaluation time point in the previous work ^27^ and the present work because the damage is already significant at this time point. RPE morphology and cell loss were evaluated by phalloidin staining for F-actin and DAPI staining for nucleus on RPE whole mounts. Treatment with NaIO_3_ induced damage in 50% of the entire RPE area in the wild-type mice. The damaged area was reduced to 25% in *Thra1^-/-^* mice (Fig. 1A-B). In contrast, there was a slight but not significant improvement in *Thra1^+/-^* mice (Fig. 1A-B). The RPE cell number in the central and middle regions was reduced by about 85% and 60%, respectively, in the wild-type mice after NaIO_3_-treatment. *Thra1^-/-^* mice showed significantly increased numbers of RPE cells, compared with that in the wild-type and *Thra1^+/-^* mice (Fig. 1C-D). Similar results were obtained in RPE nuclear number evaluations (Fig. 1D, lower panel). RPE morphology in untreated *Thra1^-/-^* mice was not different from that in the wild-type (data not shown). The protective effect of *Thra1* deletion was also observed in mice at a relatively older age. *Thra1^-/-^* and wild-type mice at 7 months received NaIO_3_ treatment and were then analyzed for RPE morphology at 2 days post-treatment. Phalloidin staining showed that the NaIO_3_ treatment caused damage in about 88% of the entire RPE area in the wild-type mice, but the damaged area was reduced to 74% in *Thra1^-/-^* mice (*p* < 0.05, Supplementary Fig. 1). The RPE cell number analysis showed similar findings (Supplementary Fig. 1).

The protective effects of *Thra1* deletion on retina/photoreceptors were demonstrated by evaluation of retinal integrity, photoreceptor number, and retinal cell death. H&E staining of the retinal cross sections showed that treatment with NaIO_3_ caused severe damage in the photoreceptor layer in the wild-type mice; manifested as a disorganized outer nuclear layer (ONL) and outer segment (OS), reduced nuclear numbers/thickness of the ONL, and shortened OS. The detrimental effects from NaIO_3_ treatment were greatly reduced in *Thrα1^-/-^* mice. After NaIO_3_ treatment, ONL thickness in the central retina of the wild-type mice was reduced by about 27%, compared with the untreated controls, and deletion of *Thra1* nearly completely prevented this reduction (Fig. 2A). Retinal morphology in untreated *Thra1^-/-^* mice was not different from that in the wild-type (Fig. 2A). Cone photoreceptor density/numbers were assessed by peanut agglutinin (PNA) labeling on retinal whole mounts. After NaIO_3_ treatment, cone density in the retinas of wild-type mice was reduced by about 37%, compared with the untreated controls. However, cone density in *Thrα1^-/-^* mice after NaIO_3_ treatment was reduced by 13% only, compared with untreated *Thrα1^-/-^* controls (Fig. 2B). Cone density in the untreated *Thra1^-/-^* mice was not different from that in the wild-type (Fig. 2B). Similar to findings in the RPE evaluations, *Thra1^+/-^* mice did not show significant protection against NaIO_3_-induced loss of cones (Fig. 2B). Retinal cell death was evaluated by terminal deoxynucleotidyltransferase dUTP nick-end labeling (TUNEL). After NaIO_3_ treatment, a large increase in the number of TUNEL-positive cells was observed in ONL areas of the retinal sections prepared from wild-type mice. However, deletion of *Thra1* nearly completely eliminated the TUNEL labeling (Fig. 2C). In this work, we also examined mRNA expression levels of *Thra1* and other THR subtypes in the RPE and retina, and their retinal localization. qRT-PCR analysis of the retinas (prepared from P30 C57BL/6J mice) showed that *Thra1*, *Thrb1,* and *Thrb2* were all expressed in the RPE and retina, and that *Thra1* was expressed at 1-2-fold higher than *Thrb1* and *Thrb2* in both tissues (Supplementary Fig. 2A). RNAscope *in situ* hybridization analysis showed the expression of *Thra1* mRNA and *Thrb* mRNA in all layers of the retina (Supplementary Fig. 2B), similar to the previous findings ^29, 30, 31^ ^38^. No difference in the expression levels of *Thrb* and *Thra1* was observed between the periphery and center of the retina.

***Deletion of Thrb*** ***protected RPE and photoreceptors from damage/cell loss induced by NaIO_3_***

We next examined the effects of THRB deletion. *Thrb^-/-^* and wild-type mice received a single injection of NaIO_3_ (30 mg/kg, i.p.) at P30, and were analyzed for RPE morphology and photoreceptor integrity at 3 days post-NaIO_3_ treatment. Phalloidin staining of the RPE whole mounts showed that treatment with NaIO_3_ caused damage in 25% of the entire RPE area in *Thrb^-/-^* mice, which was significantly lower than the 50% damaged area in the wild-type (Fig. 3A-B). RPE morphology in untreated *Thrb^-/-^* mice was not different from that in the wild-type (data not shown). The RPE cell number in the central and middle regions was reduced by about 85% and 60%, respectively, in the wild-type mice after NaIO_3_-treatment. *Thrb^-/-^* mice showed significantly increased numbers of RPE cells, compared with that in the wild-type (Fig. 3C). Similar results were obtained in RPE nuclear number evaluations (Fig. 3C-D).

The protective effects of *Thrb* deletion on retina/photoreceptors were demonstrated by evaluation of retinal integrity, photoreceptor number, and retinal cell death. The overall retinal morphology was well preserved in *Thrb^-/-^* mice after NaIO_3_ treatment, compared with that in the wild-type (Fig. 4A). After NaIO_3_ treatment, ONL thickness in the central retina of the wild-type mice was reduced by about 27%, compared with untreated controls, and deletion of *Thrb* completely prevented this reduction (Fig. 4A). Retinal morphology in untreated *Thrb^-/-^* mice was not different from that in the wild-type (Fig. 4A). PNA labeling on retinal whole mounts showed that after NaIO_3_ treatment, cone density in the wild-type mice was reduced by about 37%, compared with untreated controls. However, cone density in *Thrb^-/-^* mice after NaIO_3_ treatment was reduced by 18% only, compared with untreated *Thrb^-/-^* controls (Fig. 4B). Cone density in the untreated *Thrb^-/-^* mice was not different from that in the wild-type (Fig. 4B). Treatment with NaIO_3_ induced a large increase in the number of TUNEL-positive cells in the wild-type mice and deletion of *Thrb* significantly reduced the number of TUNEL-positive cells (Fig. 4C).

***Deletion of Thrb2*** ***protected RPE and cones but not rods from damage/cell loss induced by NaIO_3_***

We then further examined the contribution of THRB2. *Thrb2^-/-^* and wild-type mice received a single injection of NaIO_3_ (30 mg/kg, *i.p.*) at P30, and were analyzed for RPE morphology and photoreceptor integrity at 3 days post-NaIO_3_ treatment. Phalloidin staining of the RPE whole mounts showed that treatment with NaIO_3_ caused damage in 28% of the entire RPE area in *Thrb2^-/-^* mice, which was significantly lower than a 50% damaged area in the wild-type (Fig. 5A-B). RPE morphology in untreated *Thrb2^-/-^* mice was not different from that in the wild-type (data not shown). The RPE cell number in the central and middle regions was reduced by about 85% and 60%, respectively, in the wild-type mice after NaIO_3_-treatment. *Thrb2^-/-^* mice showed significantly increased numbers of RPE cells, compared with that in the wild-type (Fig. 5C-D). Similar results were obtained in RPE nuclear number evaluations (Fig. 5C-D). The protective effect of *Thrb2* deletion was also observed in mice at an old age. *Thrb2^-/-^* and wild-type mice at 17-months received NaIO_3_ treatment and were then analyzed for RPE morphology at 2 days post-treatment. Phalloidin staining showed that the NaIO_3_ treatment caused damage in about 88% of the RPE area in the wild-type mice, but the damaged area was reduced to 75% in the *Thrb2^-/-^* mice (*p* < 0.05, Supplementary Fig. 3A). The RPE cell/nuclear number analysis showed similar findings (Supplementary Fig. 3B).

The protective effects of *Thrb2* deletion on retina/photoreceptors were demonstrated by evaluation of retinal integrity, photoreceptor number, and retinal cell death. After NaIO_3_ treatment, ONL thickness in the central retina of the wild-type mice was reduced by about 27%, compared with the untreated controls. Unlike that in *Thrα1^-/-^* or *Thrb^-/-^ mice,* deletion of *Thrb2* did not prevent this reduction (Fig. 6A). Retinal morphology in untreated *Thrb2^-/-^* mice was not different from that in the wild-type (Fig. 6A). PNA labeling on retinal whole mounts showed that cone density in the wild-type mice after NaIO_3_ treatment was reduced by about 37%, compared with the untreated controls. However, cone density in *Thrb2^-/-^* mice after NaIO_3_ treatment was reduced by 16% only, compared with untreated *Thrb2^-/-^* controls (Fig. 6B). Cone density in the untreated *Thrb2^-/-^* mice was not different from that in the wild-type (Fig. 6B). Treatment with NaIO_3_ induced a large increase in the number of TUNEL-positive cells in the wild-type mice. However, deletion of *Thrb*2 did not significantly reduce the number of TUNEL-positive cells (Fig. 6C).

***Deletion of THR* *abolished/diminished NaIO_3_-induced gene expression upregulation in the RPE and retina***

To explore the mechanisms underlying THR signaling suppression-induced protection, we examined expression of the genes involved in oxidative stress responses, apoptosis/necroptosis pathways, and inflammatory responses. Wild-type, *Thrα1^-/-^* and *Thrb^-/-^* mice received a single injection of NaIO_3_ (30 mg/kg, *i.p.*) at P30, and were then analyzed for gene expression in the RPE and retinas at 1-day post-NaIO_3_ treatment. NaIO_3_ treatment significantly induced expression of these genes in the RPE (Fig. 7) and retina (Fig. 8) of the wild-type mice, and deletion of *Thrα1* or *Thrb* abolished or significantly suppressed the upregulation of the gene expression induced by NaIO_3_ (Figs. 7-8).

***Treatment with THR antagonist MLS reduced ARPE-19 cell death and hRPE cell death after NaIO_3_* *treatment***

The involvement of THR in NaIO_3_-induced RPE damage/cell loss was also examined in an *in vitro* cell culture model. The ARPE-19 cells and hRPE cells cultured in RPMI-1640 medium were treated with NaIO_3_ at 5 and 10 mM for ARPE-19 cells and 20, 30, and 40 mM for hRPE cells in the absence and presence of various concentrations of the THR antagonist MLS for 24 hours ^39, 40^, and were then analyzed for cell viability by MTS assay. MLS is a member of the methylsulfonylnitrobenzoate-containing series and it inhibits THR’s interaction with the co-activator steriod receptor coactivator 2 and antagonizes T3-activated transcription ^39^. The experimental data showed that treatment with NaIO_3_ concentration-dependently reduced viability of the ARPE-19 cells and hRPE cells, and treatment with MLS preserved viability of these cells in a concentration-dependent manner (Supplementary Fig. 4).

**Discussion**

In the previous study, we have shown that treatment with anti-thyroid drug nearly completely preserved RPE and photoreceptors from cell damage/death in mice (C57BL/6) treated with NaIO_3_ ^27^, and reversed upregulation of the genes involved in cellular stress responses and cell death. The present work evaluated the effects of deletion of *Thrα1*, *Thrb*, or *Thrb2* to understand the involvement of THRs and the contribution of the different receptor subtypes. We show that deletion of *Thrα1*, *Thrb*, or *Thrb2* protected RPE and photoreceptors from cell damage/death caused by NaIO_3_ treatment, and significantly suppressed NaIO_3_-induced gene expression alterations. In addition, treatment with THR antagonist effectively reduced NaIO_3_-induced cell death of ARPE-19 cells and hRPE cells in a cell culture system.

It is worth mentioning that a single administration of NaIO_3_ (*i.v.* or *i.p.*) selectively induces RPE/retinal damage in experimental animals (wild-type, C57BL/6) with more severe damage in the central and middle regions of the RPE/retina and less damage in the periphery. This phenomenon has been reported previously ^27, 35, 36^. The TH receptor knockout mice showed a similar phenomenon, *e.g.,* more severe damage in the center, as that in wild-type mice. It is not known at this time how the central area of the RPE/retina is more severely affected, and the phenomenon merits further investigation.

Deletion of *Thrα1* protected RPE, cones, and rods, and suppressed upregulation of the stress/death response genes in the RPE and retina. This is consistent with the broad expression of *Thrα1* in the RPE and retina ( ^30^ ^31^, also see Supplementary Fig. 2), and the functional role of this receptor subtype ^38, 41^. Of note, the heterozygous deletion of *Thrα1* did not achieve a protection, suggesting that the remaining 50% of the receptors are able to fullfil the regulation. The regulation of THRA1 in the viability of RPE and photoreceptors has never been documented. This is the first study to evalute the regulation/contribution of THRA1 signaling in the RPE and retina using mouse models. We show that THRA1 was involved in NaIO_3_-induced RPE and retinal degeneration and deletion of *Thra1* effectively protected RPE and photoreceptors.

Deletion of *Thrb* (resulting in deletion of both *Thrb1 and Thrb2*) protected RPE, rods, and cones, and suppressed upregulation of the stress/death response genes in the RPE and retina. This is consistent with the broad expression of *Thrb* in the RPE and retina (^31^ ^30^, also see Supplementary Fig. 2), and the functional role of these receptor subtypes ^38, 41^. Because THRB2 is expressed only in the cones in the retina ^29, 32, 33^, and *Thrb2* deletion has been documented in cone protection against T3-induced cell death ^14^ and in mouse models of LCA and achromatopsia ^16^, we also included *Thrb2^-/-^* mice in this study to learn more about *Thrb2* deletion-associated protection in an NaIO_3_-induced mouse model of AMD. Deletion of *Thrb2* protected RPE and cones, similar to that in mice with *Thrb* deletion. As mentioned above, the deletion of *Thrb* implies that both the THRB1 and THRB2 isoforms are missing. Based on the nature of the deletion, if both THRB1 and THRB2 isoforms are involved, we expect to see more/additive protection in *Thrb^-/-^* mice than in *Thrb2^-/-^* mice. In this study, however, we did not see additive protection in the RPE and cones in *Thrb^-/-^* mice, compared with that in *Thrb2^-/-^* mice. The protection levels against NaIO_3_-induced damage/cell death in the RPE and cones were not different between *Thrb^-/-^* mice and *Thrb2^-/-^* mice (see Figs. 3 and 5 for RPE, and Figs. 4B and 6B for cones). These observations may suggest that the protection in the RPE and cones in *Thrb^-/-^* mice is mainly mediated by deficiency of THRB2. As mentioned above, the role of THRB2 in cone opsin expression and T3-induced cone death has been well documented. The findings from the present work provided evidence showing the role of THRB2 in a different model of cone degeneration (NaIO_3_-induced cone death). The regulation of THRB/THRB2 in the RPE is little understood, and to our knowledge, this work for the first time demonstrates a role of THRB/THRB2 signaling in RPE stress/damage.

Rods were protected in *Thrb^-/-^* mice but not in *Thrb2^-/-^* mice (see Figs. 4A and 6A). This observation is consistent with the previous findings showing that THRB2 is present only in cones in the retina ^29, 32, 33^, and may suggest a critical contribution of THRB1-mediated damage of rods in the model. Interestingly, deletion of *Thrb2* protected cones and RPE despite the presence of THRB1 in these cell types. These observations may suggest a predominant contribution of THRB2-mediated damage in these cell types, although the contributions of THRB1 cannot entirely be excluded. Structurally, THRB1 and THRB2 isoforms differ in their N-terminus; codons 1-94 of THRB1 are encoded by exons that are not present in THRB2; and THRB1 is identical to THRB2 in the C-terminus ^7, 42^. How such structural differences contribute to the functional variation is not known at this time, and merits further exploration. Together, our data support the view that THRB/THRB2 signaling regulates cell damage/death of the RPE after oxidative stress challenge, and that in the retina, it is likely that THRB1 signaling regulates cell damage/death of the rods whereas THRB2 signaling regulates cell damage/death of the cones. Nevertheless, more definitive information about the role of the THRB1 isoform would come only from the use of *Thrb1^-/-^* mice, and this could be our next-step effort.

It should be noted that the protective effects of *Thra1* deletion and *Thrb2* deletion were also observed in mice at relatively older ages (see Supplementary Fig. 1 for 7-month-old *Thra1^-/-^* mice, and Supplementary Fig. 3 for 17-month-old *Thrb2^-/-^* mice). However, the protection from older *Thra1^-/-^* and *Thrb2^-/-^* mice was somewhat reduced, compared with that in the young mice (see Fig. 1A-B for 1- month-old *Thra1^-/-^* mice and Fig. 5A-B for 1- month-old *Thrb2^-/-^* mice). More severe damage in older mice after NaIO_3_ treatment, as reported previously ^27^ and as shown in the present study (see Supplementary Figs. 1, 3), may contribute to reduced protection. It might also be related to a reduced protection capacity in the older mice, which merits further investigation.

Owing to the broad regulation of TH signaling, deletion of *Thra1* or *Thrb* has effects on many tissues in experimental animals. Examples of *Thra1* deletion include impaired cardiovascular/heart function ^43^, abnormal body temperature ^44^, impaired development of brain tissues ^45^ and bone tissues ^46^, and impaired hearing system/auditory function ^47^. Examples of *Thrb* deletion include neuronal behavior defects/cerebellar defects ^48^, altered metabolism ^49^, and deafness ^50^. Deletion of *Thrb2* specifically causes cone defects ^14^. Serum T3 and T4 levels in the knockout mice are elevated by about 0.5-1.5-fold, compared with that in the wild-type mice ^14, 47, 51^. Although we cannot absolutely rule out the possibility, it is less likely that an unrelated preconditioning in these knockout mouse lines affects the retinal degeneration phenotype/the effects of the receptor deletion. The RPE and retinal morphology/integrity are not different between the untreated wild-type mice and the receptor knockout mice at the ages studied (see Figs. 2, 4, and 6).

In summary, this work demonstrated the involvement of THR signaling in cell damage/death of the RPE and photoreceptor after oxidative stress challenge. The work also provided insights into the regulation/contribution of the different THR subtypes in cell viability. Findings from this work support a role of THR signaling in the pathogenesis of AMD and the view of targeting THR signaling locally in the retinal for protection of RPE and photoreceptors in dry AMD.

**Materials and methods**

***Mice and reagents***

C57BL/6J and *Thra1*^-/- 43^ mouse lines were obtained from the Jackson Laboratory, Thrb^-/- 51^ and Thrb2^-/-^ ^14^ mouse lines were provided by Dr. Douglas Forrest (National Institute of Diabetes and Digestive and Kidney Diseases, NIH). Mice were maintained under cyclic light (12-h light - dark) conditions. Cage illumination was 7-foot-candle during the light cycle. All animal maintenance and experiments were approved by the local Institutional Animal Care and Use Committee (University of Oklahoma Health Sciences Center) and conformed to the guidelines on the care and use of animals adopted by the Society for Neuroscience and the Association for Research in Vision and Ophthalmology. Mice of either sex were used in the experiments. Mice were randomly assigned, within a litter, for the drug treatment or vehicle/untreated experiments; littermate controls were used whenever possible; and no animals were excluded from the analysis. No blinding was carried out for animal experiments.

Alexa Fluor® 594 phalloidin (Catalog#: A12381) and Alexa Fluor® 488 donkey anti-rabbit IgG (Catalog#: A21206) were purchased from Life Technologies; DAPI (4,6-Diamidino-2-phenylindole, Catalog#: D9542), NaIO_3_ (Catalog#: S4007) were purchased from Millipore Sigma; biotinylated PNA (Catalog#: B-1075) was purchased from Vector Labs. MLS000389544 (MLS) was purchased from Sigma-Aldrich (Cat#: 573965-48-7).

***Treatment of NaIO_3_***

NaIO_3_ treatment was performed as described previously ^37^. Briefly, mice received a single injection of NaIO_3_ (30 mg/kg, *i.p.*) at P30 or other ages as indicated, and were then analyzed for RPE and photoreceptor damage/cell death at 3 days post-NaIO_3_ injection.

***Eye preparation, immunofluorescence labeling, confocal microscopy, and retinal morphometric analysis***

The RPE whole mounts were prepared for immunofluorescence labeling. Briefly, eyes were enucleated and fixed in 4% paraformaldehyde (PFA; Polysciences, Inc.) for 1 h at room temperature, followed by removal of the cornea, lens, muscles, and retina. The RPE sheets (the sclera-choroid-RPE sheets) were then fixed in 4% PFA for another 1 hour at room temperature, followed by wash (PBS, 5 min 3x) and blocking with 10% FBS in 0.5% Triton X-100 in PBS for 1 hour at room temperature. The RPE sheets were then stained with Alexa Fluor® 594 phalloidin (1:40) for 30-45 min at room temperature and DAPI (1 ng/mL) for another 30 min at room temperature, followed by wash (PBS, 5 min 2x). The RPE whole mounts were made by transferring the sheets onto the slides, followed by mounting with Hard medium (H-1500, Vector Laboratories).

The retinal whole mounts and cross sections were prepared for immunofluorescence labeling, as described previously ^12^. For retinal whole mount preparations, eyes were enucleated, marked at the superior pole with a green dye, and fixed in 4% PFA for 30-60 min at room temperature, followed by removal of the cornea and lens. The eyes were then fixed in 4% PFA in PBS for 4–6 h at room temperature, and retinas were isolated and the superior portion was marked for orientation with a small cut. For retinal paraffin sections, eyes were enucleated (the superior portion of the cornea was marked with green dye prior to enucleation) and fixed in Prefer (Anatech Ltd.) for 25-30 min at room temperature. Sections (5-µm thickness) passing vertically through the retina (along the vertical meridian passing through the optic nerve head) were prepared using a Leica microtome (Leica Biosystems), and were used for hematoxylin and eosin (H&E) staining. For retinal cryosections, eyes were fixed in 4% PFA for 1 hour, and the cornea and lens were then removed, followed by fixing the eye-cups in 4% PFA for 3 hours. The eye-cups were then soaked in graded concentrations of sucrose overnight at 4°C. After embedded with an optimal cutting temperature (OCT) compound, 5-μm retinal sections were prepared using a Thermo Scientific CryoStar NX70 Cryostat. Prior to blocking on retinal sections, antigen retrieval was performed in 10 mM sodium citrate buffer (pH 6.0) for 30 min in a 70 °C water bath. Retinal whole mounts or sections were blocked with Hanks’ balanced salt solution containing 5% BSA and 0.5% Triton X-100 for 1 h at room temperature or overnight at 4 °C. Peanut agglutinin (PNA) immunohistochemistry was performed using biotinylated PNA (1:250) and streptavidin-Cy3 (1:500).

Low magnification images were taken under the Olympus MVX10 dissection microscope equipped with Image-Pro 6.3 software (Media Cybernetics, Inc.). High magnification images were taken with 60X or 100x objectives on the FV1000 confocal laser-scanning microscope equipped with FluoView imaging software (Olympus, Melville). ImageJ software (https://imagej.net/) was used to analyze the damaged area on the RPE whole mounts. For quantification of RPE cell numbers and RPE nuclear numbers, images from four quadrants in the central, middle and peripheral regions were counted and normalized to the number in one square millimeter. Evaluation of cone density on retinal whole mounts was performed as described previously^12, 52^. For retinal morphometric analysis, retinal cross sections stained with H&E were used for morphometric analysis to evaluate ONL integrity/rod survival, as described previously ^12, 53^.

***TUNEL assays***

Terminal deoxynucleotidyltransferase dUTP nick-end labeling (TUNEL) was performed on paraffin-embedded retinal sections, using an *in situ* cell death fluorescein detection kit (Sigma-Aldrich, Catalog#: 11684795910), as described previously ^54^. Immunofluorescence signals were imaged using an Olympus FV1000 confocal laser-scanning microscope. TUNEL-positive cells in the outer nuclear layer passing through the optic nerve were counted and averaged from at least three sections per eye, from 3-5 mice per condition.

***RNA isolation and quantitative real-time PCR***

The mouse RPE cells were isolated as described ^55^. Total RNA preparation and reverse transcription were performed as described previously ^56^. The gene encoding the mouse hypoxanthine guanine phosphoribosyl transferase 1 (*Hprt1*) was included as an internal control. Supplemental Table 1 shows the primers used. The quantitative real-time PCR (qRT-PCR) assays were performed using a real-time PCR detection system (iCycler; Bio-Rad Laboratories, Hercules, CA, USA), and the relative gene expression value was calculated based on the ΔΔCt method, as described previously ^56^.

**RNAscope *in situ* hybridization**

RNAscope *in situ* hybridization was applied to examine the expression/localization of mRNA levels of *Thra1* and *Thrb* in the retina, as described ^57, 58^. The assays were conducted using RNAscope® 2.5 HD Detection Reagent-Red Kit (Advanced Cell Diagnostics, Catalog #: 322360), as per manufacturer’s instructions. Briefly, cryosections of the mouse retinas (5-μm) were hybridized with the target probes for *Thra1* (Catalog #: 531731, Advanced Cell Diagnostics) and for *Thrb* (Catalog #: 544331, Advanced Cell Diagnostics) at 40 °C for 2 hours, with negative and positive controls (Catalog #: 31004 and Catalog #: 313911). *Thra1* probe targets the region between 1836 and 2336 of the *Thra1* mRNA (NM_178060.4). *Thrb* probe targets the region between 32 and 461 of the *Thrb* mRNA (NM_001113417.1), with potential detection of both *Thrb1* and *Thrb2*. The slides were then counterstained with 50% hematoxylin blued with 0.02% ammonia water, dried, and mounted, and images were acquired using an Olympus microscope.

***ARPE-19 and hRPE cell culture and drug treatment***

ARPE-19 (ATCC, Manassas, VA) cells and hRPE cells (kindly provided by Dr. Goldis Malek at Duke University) were cultured in RPMI-1640 medium (ATCC) with 10% FBS, as described ^59, 60^. To examine the effects of THR antagonist MLS on NaIO_3_-induced cell death, cells were cultured in RPMI-1640 medium with 10% FBS for 24 hours and were then treated with NaIO_3_ at various concentrations in the absence and presence of MLS for another 24 hours, followed by MTS assay for evaluation of cell viability.

***MTS assay***

# The cell viability/proliferation assay (MTS) was performed using One Solution Cell Proliferation Assay kit (CellTiter 96® AQueous One Solution, Promega, Madison, WI, USA), as per manufacture’s instruction. The results of MTS assay were obtained by measuring absorbance at 490 nm with a fluorescence plate reader (Molecular Devices, Sunnyvale, CA, USA). All assays were performed in triplicate and experiments were repeated three times.

***Statistical analysis***

Results are expressed as means ± *SEM* of number of mice. Power analysis was performed to choose the sample size. The analysis indicates that a sample size of 3-6 mice/group for evaluations of retinal degeneration in the mouse retinas will provide at least 80% power (1-β) for a two-sided, two-sample t-test at a 0.05 alpha level. One-way ANOVA was used to analyze for significance within sets of data, and two-tailed Student’s *t*-test was used for differences between two groups of data. Differences were considered statistically significant when *p*< 0.05. Statistical tests for every figure are justified as appropriate. Data were analyzed and graphed using GraphPad Prism® software (GraphPad Software, San Diego, CA).

**Data availability**

All data needed to evaluate the conclusions in the paper are present in the paper. Additional data related to this paper may be requested from the corresponding author.

**Acknowledgments**

This work was supported by the BrightFocus Foundation grant M2018107, the Oklahoma Center for the Advancement of Science and Technology, the Presbyterian Health Foundation, and the NEI grant P30EY021725.

We thank Dr. Douglas Forrest for providing Thrb^-/-^ and Thrb2^-/-^ mouse lines. We thank Dr. Goldis Malek for providing hRPE cell lines. We thank the Imaging Core Facility and the Histology Core Facility of the Department of Cell Biology at the University of Oklahoma Health Sciences Center for technical assistance.

###

### **Competing interests**

##### The authors declare no competing interests.

**Supplementary information is available at *Cell Death and Disease*’s website.**

**References**

1. Ambati J, Fowler BJ. Mechanisms of age-related macular degeneration. *Neuron* 2012, **75**(1)**:** 26-39.

2. Handa JT, Bowes Rickman C, Dick AD, Gorin MB, Miller JW, Toth CA*, et al.* A systems biology approach towards understanding and treating non-neovascular age-related macular degeneration. *Nature communications* 2019, **10**(1)**:** 3347.

3. Pool FM, Kiel C, Serrano L, Luthert PJ. Repository of proposed pathways and protein-protein interaction networks in age-related macular degeneration. *NPJ Aging Mech Dis* 2020, **6:** 2-2.

4. Friedman DS, O'Colmain BJ, Munoz B, Tomany SC, McCarty C, de Jong PT*, et al.* Prevalence of age-related macular degeneration in the United States. *Arch Ophthalmol* 2004, **122**(4)**:** 564-572.

5. Cruz-Guilloty F, Perez VL. Molecular medicine: Defence against oxidative damage. *Nature* 2011, **478**(7367)**:** 42-43.

6. Bowes Rickman C, Farsiu S, Toth CA, Klingeborn M. Dry age-related macular degeneration: mechanisms, therapeutic targets, and imaging. *Invest Ophthalmol Vis Sci* 2013, **54**(14)**:** ORSF68-80.

7. Brent GA. Mechanisms of thyroid hormone action. *J Clin Invest* 2012, **122**(9)**:** 3035-3043.

8. Cheng SY, Leonard JL, Davis PJ. Molecular aspects of thyroid hormone actions. *Endocr Rev* 2010, **31**(2)**:** 139-170.

9. Forrest D, Reh TA, Rusch A. Neurodevelopmental control by thyroid hormone receptors. *Curr Opin Neurobiol* 2002, **12**(1)**:** 49-56.

10. Roberts MR, Srinivas M, Forrest D, Morreale de Escobar G, Reh TA. Making the gradient: thyroid hormone regulates cone opsin expression in the developing mouse retina. *Proc Natl Acad Sci USA* 2006, **103:** 6218-6223.

11. Ng L, Lyubarsky A, Nikonov SS, Ma M, Srinivas M, Kefas B*, et al.* Type 3 deiodinase, a thyroid-hormone-inactivating enzyme, controls survival and maturation of cone photoreceptors. *J Neurosci* 2010, **30**(9)**:** 3347-3357.

12. Ma H, Thapa A, Morris L, Redmond TM, Baehr W, Ding XQ. Suppressing thyroid hormone signaling preserves cone photoreceptors in mouse models of retinal degeneration. *Proc Natl Acad Sci U S A* 2014, **111**(9)**:** 3602-3607.

13. Yang F, Ma H, Belcher J, Butler MR, Redmond TM, Boye SL*, et al.* Targeting iodothyronine deiodinases locally in the retina is a therapeutic strategy for retinal degeneration. *FASEB J* 2016, **30**(12)**:** 4313-4325.

14. Ng L. A thyroid hormone receptor that is required for the development of green cone photoreceptors. *Nature Genet* 2001, **27:** 94-98.

15. Yang F, Ma H, Butler MR, Ding XQ. Deficiency of type 2 iodothyronine deiodinase reduces necroptosis activity and oxidative stress responses in retinas of Leber congenital amaurosis model mice. *FASEB J* 2018**:** fj201800484RR.

16. Ma HW, Yang F, Butler MR, Belcher J, Redmond TM, Placzek AT*, et al.* Inhibition of thyroid hormone receptor locally in the retina is a therapeutic strategy for retinal degeneration. *FASEB Journal* 2017, **31**(8)**:** 3425-3438.

17. Chaker L, Buitendijk GH, Dehghan A, Medici M, Hofman A, Vingerling JR*, et al.* Thyroid function and age-related macular degeneration: a prospective population-based cohort study--the Rotterdam Study. *BMC Med* 2015, **13:** 94.

18. Gopinath B, Liew G, Kifley A, Mitchell P. Thyroid Dysfunction and Ten-Year Incidence of Age-Related Macular Degeneration. *Invest Ophthalmol Vis Sci* 2016, **57**(13)**:** 5273-5277.

19. Age-Related Eye Disease Study Research G. Risk factors associated with age-related macular degeneration. A case-control study in the age-related eye disease study: Age-Related Eye Disease Study Report Number 3. *Ophthalmology* 2000, **107**(12)**:** 2224-2232.

20. Chatziralli I, Mitropoulos PG, Niakas D, Labiris G. Thyroidopathy and Age-Related Macular Degeneration: Is There Any Correlation? *Biomed Hub* 2017, **2**(454706).

21. Lin SY, Hsu WH, Lin CL, Lin CC, Lin JM, Chang YL*, et al.* Evidence for an Association between Macular Degeneration and Thyroid Cancer in the Aged Population. *Int. J. Environ. Res. Public Health* 2018, **15**(5): 902.

22. Ceresini G, Lauretani F, Maggio M, Ceda GP, Morganti S, Usberti E*, et al.* Thyroid function abnormalities and cognitive impairment in elderly people: results of the Invecchiare in Chianti study. *J Am Geriatr Soc* 2009, **57**(1)**:** 89-93.

23. Kalmijn S, Mehta KM, Pols HA, Hofman A, Drexhage HA, Breteler MM. Subclinical hyperthyroidism and the risk of dementia. The Rotterdam study. *Clin Endocrinol (Oxf)* 2000, **53**(6)**:** 733-737.

24. Hosoda L, Adachi-Usami E, Mizota A, Hanawa T, Kimura T. Early effects of sodium iodate injection on ERG in mice. *Acta Ophthalmol (Copenh)* 1993, **71**(5)**:** 616-622.

25. Chowers G, Cohen M, Marks-Ohana D, Stika S, Eijzenberg A, Banin E*, et al.* Course of Sodium Iodate-Induced Retinal Degeneration in Albino and Pigmented Mice. *Invest Ophthalmol Vis Sci* 2017, **58**(4)**:** 2239-2249.

26. Zhao C, Yasumura D, Li X, Matthes M, Lloyd M, Nielsen G*, et al.* mTOR-mediated dedifferentiation of the retinal pigment epithelium initiates photoreceptor degeneration in mice. *J Clin Invest* 2011, **121**(1)**:** 369-383.

27. Ma H, Yang F, Ding XQ. Inhibition of thyroid hormone signaling protects retinal pigment epithelium and photoreceptors from cell death in a mouse model of age-related macular degeneration. *Cell Death Dis* 2020, **11**(1)**:** 24, https://doi.org/10.1038/s41419-019-2216-7.

28. Flamant F, Baxter JD, Forrest D, Refetoff S, Samuels H, Scanlan TS*, et al.* International Union of Pharmacology. LIX. The pharmacology and classification of the nuclear receptor superfamily: thyroid hormone receptors. *Pharmacol Rev* 2006, **58**(4)**:** 705-711.

29. Ng L, Ma M, Curran T, Forrest D. Developmental expression of thyroid hormone receptor β2 protein in cone photoreceptors in the mouse. *Neuroreport* 2009, **20:** 627-631.

30. Trimarchi JM, Harpavat S, Billings NA, Cepko CL. Thyroid hormone components are expressed in three sequential waves during development of the chick retina. *BMC Dev Biol* 2008, **8:** 101.

31. Volkov LI, Kim-Han JS, Saunders LM, Poria D, Hughes AEO, Kefalov VJ*, et al.* Thyroid hormone receptors mediate two distinct mechanisms of long-wavelength vision. *Proc Natl Acad Sci U S A* 2020, **117**(26)**:** 15262-15269.

32. Applebury ML, Farhangfar F, Glosmann M, Hashimoto K, Kage K, Robbins JT*, et al.* Transient expression of thyroid hormone nuclear receptor TRbeta2 sets S opsin patterning during cone photoreceptor genesis. *Dev Dyn* 2007, **236**(5)**:** 1203-1212.

33. Jones I, Ng L, Liu H, Forrest D. An intron control region differentially regulates expression of thyroid hormone receptor β2 in the cochlea, pituitary, and cone photoreceptors. *Mol Endocrinol* 2007, **21:** 1108-1119.

34. Glaschke A, Weiland J, Del Turco D, Steiner M, Peichl L, Glosmann M. Thyroid hormone controls cone opsin expression in the retina of adult rodents. *J Neurosci* 2011, **31**(13)**:** 4844-4851.

35. Machalinska A, Lubinski W, Klos P, Kawa M, Baumert B, Penkala K*, et al.* Sodium iodate selectively injuries the posterior pole of the retina in a dose-dependent manner: morphological and electrophysiological study. *Neurochem Res* 2010, **35**(11)**:** 1819-1827.

36. Kiuchi K, Yoshizawa K, Shikata N, Moriguchi K, Tsubura A. Morphologic characteristics of retinal degeneration induced by sodium iodate in mice. *Curr Eye Res* 2002, **25**(6)**:** 373-379.

37. Wang J, Iacovelli J, Spencer C, Saint-Geniez M. Direct effect of sodium iodate on neurosensory retina. *Invest Ophthalmol Vis Sci* 2014, **55**(3)**:** 1941-1953.

38. Duncan KG, Bailey KR, Baxter JD, Schwartz DM. The human fetal retinal pigment epithelium: A target tissue for thyroid hormones. *Ophthalmic Res* 1999, **31**(6)**:** 399-406.

39. Hwang JY, Huang W, Arnold LA, Huang R, Attia RR, Connelly M*, et al.* Methylsulfonylnitrobenzoates, a new class of irreversible inhibitors of the interaction of the thyroid hormone receptor and its obligate coactivators that functionally antagonizes thyroid hormone. *J Biol Chem* 2011, **286**(14)**:** 11895-11908.

40. Johnson RL, Hwang JY, Arnold LA, Huang R, Wichterman J, Augustinaite I*, et al.* A quantitative high-throughput screen identifies novel inhibitors of the interaction of thyroid receptor beta with a peptide of steroid receptor coactivator 2. *J Biomol Screen* 2011, **16**(6)**:** 618-627.

41. Minakhina S, Bansal S, Zhang A, Brotherton M, Janodia R, De Oliveira V*, et al.* A Direct Comparison of Thyroid Hormone Receptor Protein Levels in Mice Provides Unexpected Insights into Thyroid Hormone Action. *Thyroid* 2020, **30**(8)**:** 1193-1204.

42. Bassett JH, Williams GR. The skeletal phenotypes of TRalpha and TRbeta mutant mice. *J Mol Endocrinol* 2009, **42**(4)**:** 269-282.

43. Wikstrom L, Johansson C, Salto C, Barlow C, Campos Barros A, Baas F*, et al.* Abnormal heart rate and body temperature in mice lacking thyroid hormone receptor alpha 1. *EMBO J* 1998, **17**(2)**:** 455-461.

44. Golozoubova V, Gullberg H, Matthias A, Cannon B, Vennstrom B, Nedergaard J. Depressed thermogenesis but competent brown adipose tissue recruitment in mice devoid of all hormone-binding thyroid hormone receptors. *Mol Endocrinol* 2004, **18**(2)**:** 384-401.

45. Krieger TG, Moran CM, Frangini A, Visser WE, Schoenmakers E, Muntoni F*, et al.* Mutations in thyroid hormone receptor alpha1 cause premature neurogenesis and progenitor cell depletion in human cortical development. *Proc Natl Acad Sci U S A* 2019, **116**(45)**:** 22754-22763.

46. Kindblom JM, Gothe S, Forrest D, Tornell J, Tornell J, Vennstrom B*, et al.* GH substitution reverses the growth phenotype but not the defective ossification in thyroid hormone receptor alpha 1-/-beta-/- mice. *J Endocrinol* 2001, **171**(1)**:** 15-22.

47. Cordas EA, Ng L, Hernandez A, Kaneshige M, Cheng SY, Forrest D. Thyroid hormone receptors control developmental maturation of the middle ear and the size of the ossicular bones. *Endocrinology* 2012, **153**(3)**:** 1548-1560.

48. Hashimoto K, Curty FH, Borges PP, Lee CE, Abel ED, Elmquist JK*, et al.* An unliganded thyroid hormone receptor causes severe neurological dysfunction. *Proc Natl Acad Sci U S A* 2001, **98**(7)**:** 3998-4003.

49. Grover GJ, Mellstrom K, Ye L, Malm J, Li YL, Bladh LG*, et al.* Selective thyroid hormone receptor-beta activation: a strategy for reduction of weight, cholesterol, and lipoprotein (a) with reduced cardiovascular liability. *Proc Natl Acad Sci U S A* 2003, **100**(17)**:** 10067-10072.

50. Forrest D, Erway LC, Ng L, Altschuler R, Curran T. Thyroid hormone receptor beta is essential for development of auditory function. *Nat Genet* 1996, **13**(3)**:** 354-357.

51. Forrest D, Hanebuth E, Smeyne RJ, Everds N, Stewart CL, Wehner JM*, et al.* Recessive resistance to thyroid hormone in mice lacking thyroid hormone receptor beta: evidence for tissue-specific modulation of receptor function. *EMBO J* 1996, **15**(12)**:** 3006-3015.

52. Xu J, Morris L, Thapa A, Ma H, Michalakis S, Biel M*, et al.* cGMP accumulation causes photoreceptor degeneration in CNG channel deficiency: evidence of cGMP cytotoxicity independently of enhanced CNG channel function. *J Neurosci* 2013, **33**(37)**:** 14939-14948.

53. Xu J, Morris LM, Michalakis S, Biel M, Fliesler SJ, Sherry DM*, et al.* CNGA3 deficiency affects cone synaptic terminal structure and function and leads to secondary rod dysfunction and degeneration. *Invest Ophthalmol Vis Sci* 2012, **53**(3)**:** 1117-1129.

54. Ma H, Butler MR, Thapa A, Belcher J, Yang F, Baehr W*, et al.* cGMP/Protein Kinase G Signaling Suppresses Inositol 1,4,5-Trisphosphate Receptor Phosphorylation and Promotes Endoplasmic Reticulum Stress in Photoreceptors of Cyclic Nucleotide-gated Channel-deficient Mice. *J Biol Chem* 2015, **290**(34)**:** 20880-20892.

55. Wang CXZ, Zhang K, Aredo B, Lu H, Ufret-Vincenty RL. Novel method for the rapid isolation of RPE cells specifically for RNA extraction and analysis. *Exp Eye Res* 2012, **102C**.

56. Ma H, Thapa A, Morris LM, Michalakis S, Biel M, Frank MB*, et al.* Loss of cone cyclic nucleotide-gated channel leads to alterations in light response modulating system and cellular stress response pathways: a gene expression profiling study. *Hum Mol Genet* 2013, **22**(19)**:** 3906-3919.

57. Stempel AJ, Morgans CW, Stout JT, Appukuttan B. Simultaneous visualization and cell-specific confirmation of RNA and protein in the mouse retina. *Mol Vis* 2014, **20:** 1366-1373.

58. Wang F, Flanagan J, Su N, Wang LC, Bui S, Nielson A*, et al.* RNAscope: a novel in situ RNA analysis platform for formalin-fixed, paraffin-embedded tissues. *J Mol Diagn* 2012, **14**(1)**:** 22-29.

59. Dwyer MA, Kazmin D, Hu P, McDonnell DP, Malek G. Research resource: nuclear receptor atlas of human retinal pigment epithelial cells: potential relevance to age-related macular degeneration. *Mol Endocrinol* 2011, **25**(2)**:** 360-372.

60. Hanus J, Zhang H, Wang Z, Liu Q, Zhou Q, Wang S. Induction of necrotic cell death by oxidative stress in retinal pigment epithelial cells. *Cell Death Dis* 2013, **4:** e965.

**Figure Legends**

**Figure 1. Deletion of *Thrα1* protected RPE from cell damage/loss induced by NaIO_3_.** RPE morphology and cell loss were evaluated by phalloidin staining for F-actin and DAPI staining for nucleus on RPE whole mounts prepared from *Thrα1^-/-^*, *Thra1^+/-^*, and wild-type mice at 3 days post-NaIO_3_ injection. **A-B.** Shown are representative low magnification images of phalloidin staining of the damaged area in the RPE (**A**) and corresponding quantitative analysis of the damaged area (**B**). **C-D.** Shown are representative high magnification images of phalloidin staining and DAPI labeling taken at different regions of the RPE (**C**) and corresponding quantitative analysis of RPE cell numbers and RPE nuclear numbers (**D**). Data are represented as means ± *SEM* for 5-22 mice per group (***p* < 0.01, ****p* < 0.001; compared with wild-type mice treated with NaIO_3_).

**Figure 2. Deletion of *Thrα1* protected rod and cone photoreceptors from cell loss induced by NaIO_3_.** Retinal morphology, photoreceptor layer integrity, and loss of photoreceptors were evaluated by light microscope and morphometric analysis in *Thrα1^-/-^*, *Thrα1^+/-^*, and wild-type mice at 3 days post-NaIO_3_ injection. Cone density was evaluated by PNA labeling on retinal whole mounts, and photoreceptor apoptosis was evaluated by TUNEL assay. **A.** Shown are representative light microscopic images of H&E stained retinal sections, and corresponding quantitative analysis of ONL thickness in the dorsal and ventral regions. RPE, retinal pigment epithelial; ONL, outer nuclear layer; INL, inner nuclear layer; IPL, inner plexiform layer. **B.** Shown are representative confocal images of PNA labeling on retinal whole mounts, and corresponding quantitative analysis. **C.** Shown are representative confocal images of TUNEL labeling on retinal sections and corresponding quantitative analysis. Data are represented as means ± *SEM* for 3-17 mice per group (**p* < 0.05, ***p* < 0.01, ****p* <0.001; compared with wild-type mice treated with NaIO_3_).

**Figure 3. Deletion of *Thrb*** **protected RPE from cell damage/loss induced by NaIO_3_.** RPE morphology and cell loss were evaluated by phalloidin staining for F-actin and DAPI staining for nucleus on RPE whole mounts prepared from *Thrb^-/-^* and wild-type mice at 3 days post-NaIO_3_ injection. **A-B.** Shown are representative low magnification images of phalloidin staining of the damaged area in the RPE (**A**) and corresponding quantitative analysis of the damaged area (**B**). **C-D.** Shown are representative high magnification images of phalloidin staining and DAPI labeling taken at different regions of the RPE (**C**) and corresponding quantitative analysis of RPE cell numbers and RPE nuclear numbers (**D**). Data are represented as means ± *SEM* for 5-22 mice per group (***p* < 0.01, ****p* < 0.001; compared with wild-type mice treated with NaIO_3_).

**Figure 4. Deletion of *Thrb* protected rod and cone photoreceptors from cell loss induced by NaIO_3_.** Retinal morphology, photoreceptor layer integrity, and loss of photoreceptors were evaluated by light microscope and morphometric analysis in *Thrb^-/-^* and wild-type mice at 3 days post-NaIO_3_ injection. Cone density was evaluated by PNA labeling on retinal whole mounts, and photoreceptor apoptosis was evaluated by TUNEL assay. **A.** Shown are representative light microscopic images of H&E stained retinal sections, and corresponding quantitative analysis of ONL thickness in the dorsal and ventral regions. RPE, retinal pigment epithelial; ONL, outer nuclear layer; INL, inner nuclear layer; IPL, inner plexiform layer. **B.** Shown are representative confocal images of PNA labeling on retinal whole mounts, and corresponding quantitative analysis. **C.** Shown are representative confocal images of TUNEL labeling on retinal sections and corresponding quantitative analysis. Data are represented as means ± *SEM* for 4-17 mice per group (**p* < 0.05, ***p* < 0.01, ****p* <0.001; compared with wild-type mice treated with NaIO_3_).

**Figure 5. Deletion of *Thrb2*** **protected RPE from cell damage/loss induced by NaIO_3_.** RPE morphology and cell loss were evaluated by phalloidin staining for F-actin and DAPI staining for nucleus on RPE whole mounts prepared from *Thrb2^-/-^* and wild-type mice at 3 days post-NaIO_3_ injection. **A-B.** Shown are representative low magnification images of phalloidin staining of the damaged area in the RPE (**A**) and corresponding quantitative analysis of the damaged area (**B**). **C-D.** Shown are representative high magnification images of phalloidin staining and DAPI labeling taken at different regions of the RPE (**C**) and corresponding quantitative analysis of RPE cell numbers and RPE nuclear numbers (**D**). Data are represented as means ± *SEM* for 5-22 mice per group (***p* < 0.01, ****p* < 0.001; compared with wild-type mice treated with NaIO_3_).

**Figure 6. Deletion of *Thrb2*** **protected cones but not rods from cell loss induced by NaIO_3_.** Retinal morphology, photoreceptor layer integrity, and loss of photoreceptors were evaluated by light microscope and morphometric analysis in *Thrb2^-/-^* and wild-type mice at 3 days post-NaIO_3_ injection. Cone density was evaluated by PNA labeling on retinal whole mounts, and photoreceptor apoptosis was evaluated by TUNEL assay. **A.** Shown are representative light microscopic images of H&E stained retinal sections, and corresponding quantitative analysis of ONL thickness in the dorsal and ventral regions. RPE, retinal pigment epithelial; ONL, outer nuclear layer; INL, inner nuclear layer; IPL, inner plexiform layer. **B.** Shown are representative confocal images of PNA labeling on retinal whole mounts, and corresponding quantitative analysis. **C.** Shown are representative confocal images of TUNEL labeling on retinal sections and corresponding quantitative analysis. Data are represented as means ± *SEM* for 4-17 mice per group (**p* < 0.05, ****p* <0.001; compared with wild-type mice treated with NaIO_3_).

**Figure 7.** **Deletion of *Thrα1 and Thrb* reduced NaIO_3_-induced gene expression upregulation in the RPE.** Expression levels of the genes involved in cellular stress responses and death signaling in the RPE were examined by qRT-PCR in *Thrα1^-/-^*, *Thrb^-/-^*, and wild-type mice at 1 days post-NaIO_3_ injection. Shown are expression levels of the genes involved in oxidative stress responses (**A**), inflammatory responses (**B**), necroptosis pathways (**C**), and apoptosis (**B**). Data are represented as means ± *SEM* for 3-4 assays using RPE prepared from 4-5 mice per group (**p* < 0.05, ***p* < 0.01; compared with their respective untreated controls).

**Figure 8.** **Deletion of *Thrα1 and Thrb* reduced NaIO_3_-induced gene expression upregulation in the retina.** Expression levels of the genes involved in cellular stress responses and death signaling in the retina were examined by qRT-PCR in *Thrα1^-/-^*, *Thrb^-/-^*, and wild-type mice at 1 days post-NaIO_3_ injection. Shown are expression levels of the genes involved in oxidative stress responses (**A**), inflammatory responses (**B**), necroptosis pathways (**C**), and apoptosis (**B**). Data are represented as means ± *SEM* for 3-4 assays using retina prepared from 4-5 mice per group (**p* < 0.05, ***p* < 0.01, and ****p* < 0.001; compared with their respective untreated controls).

Figure 1


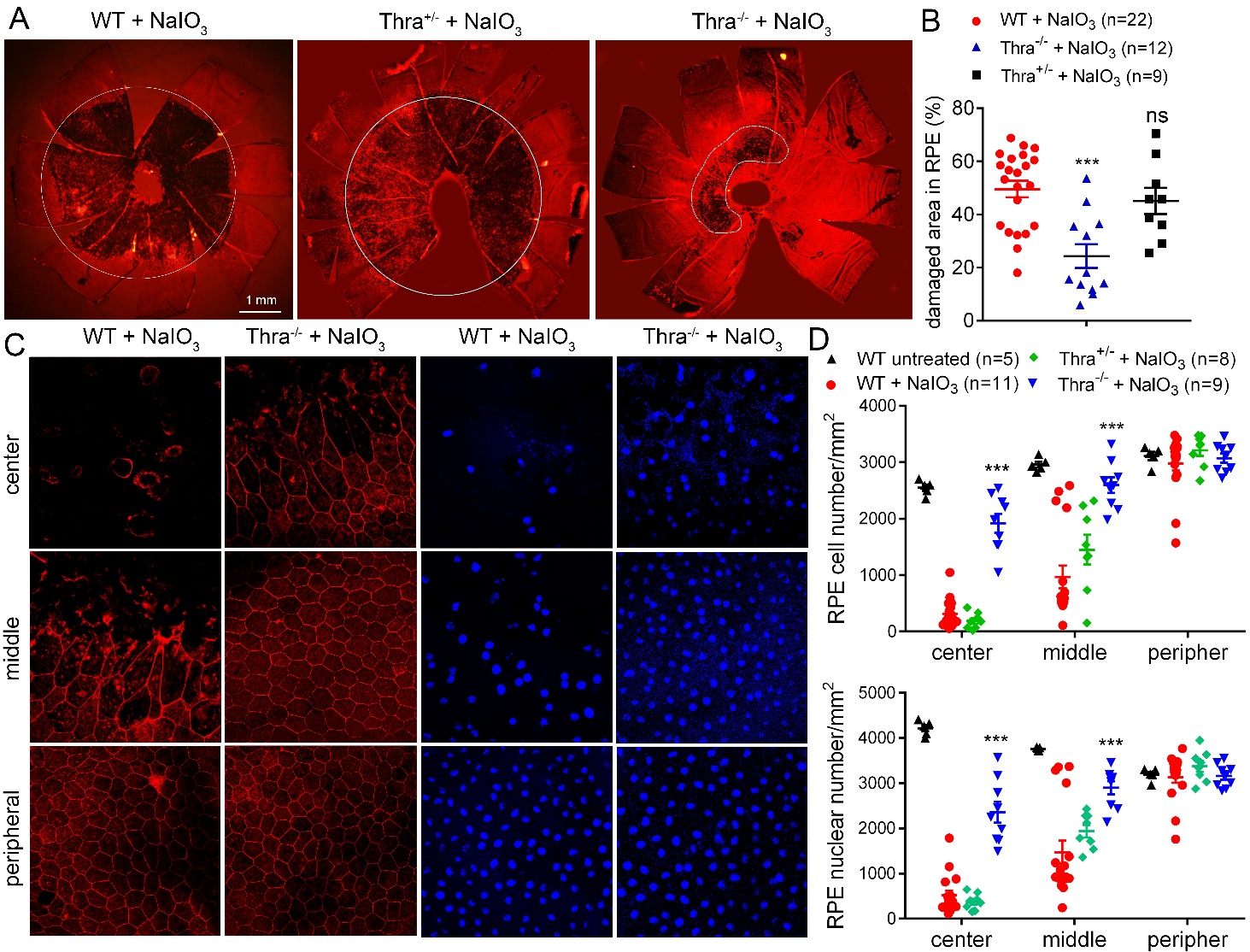


Figure 2


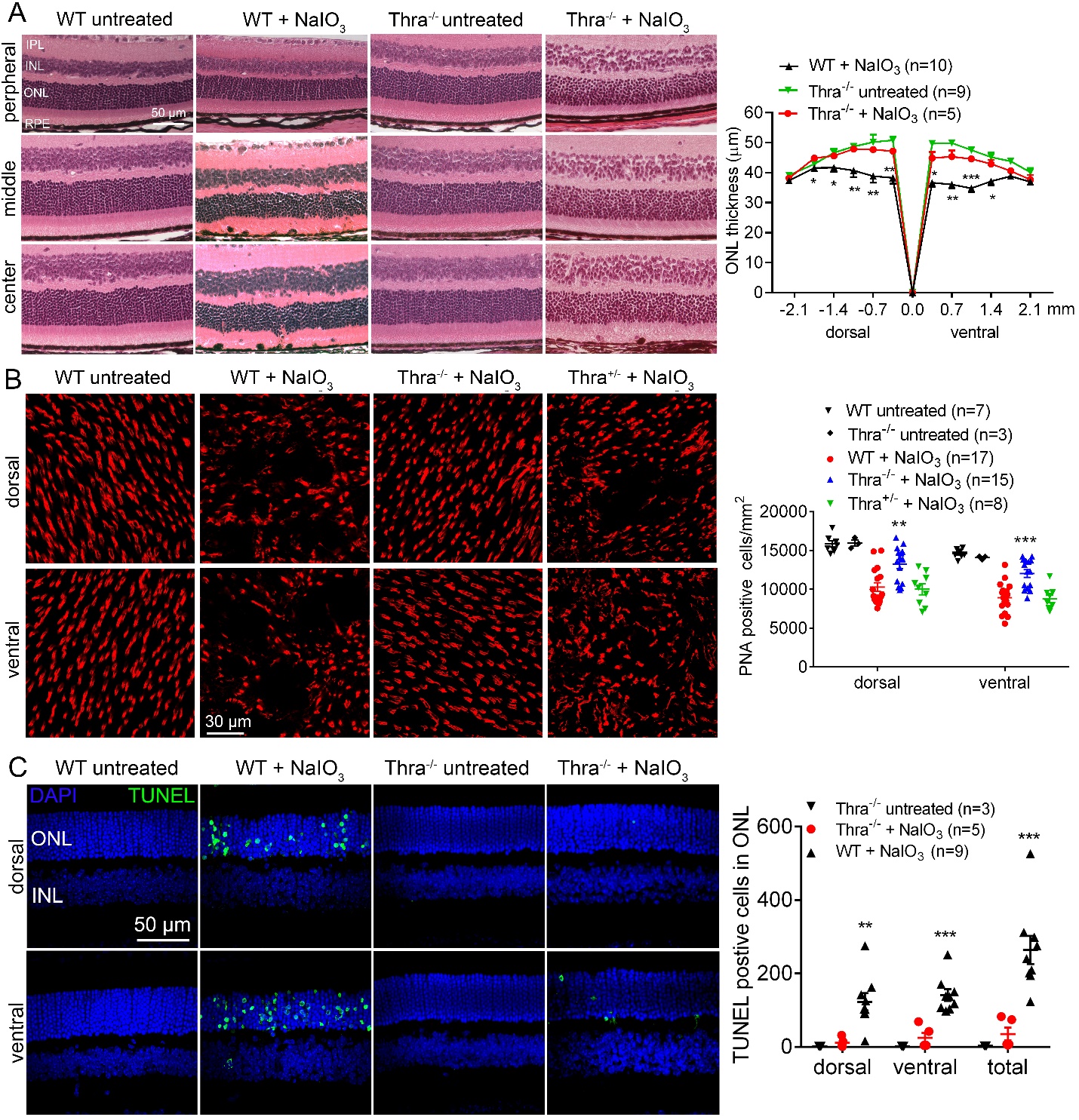


Figure 3


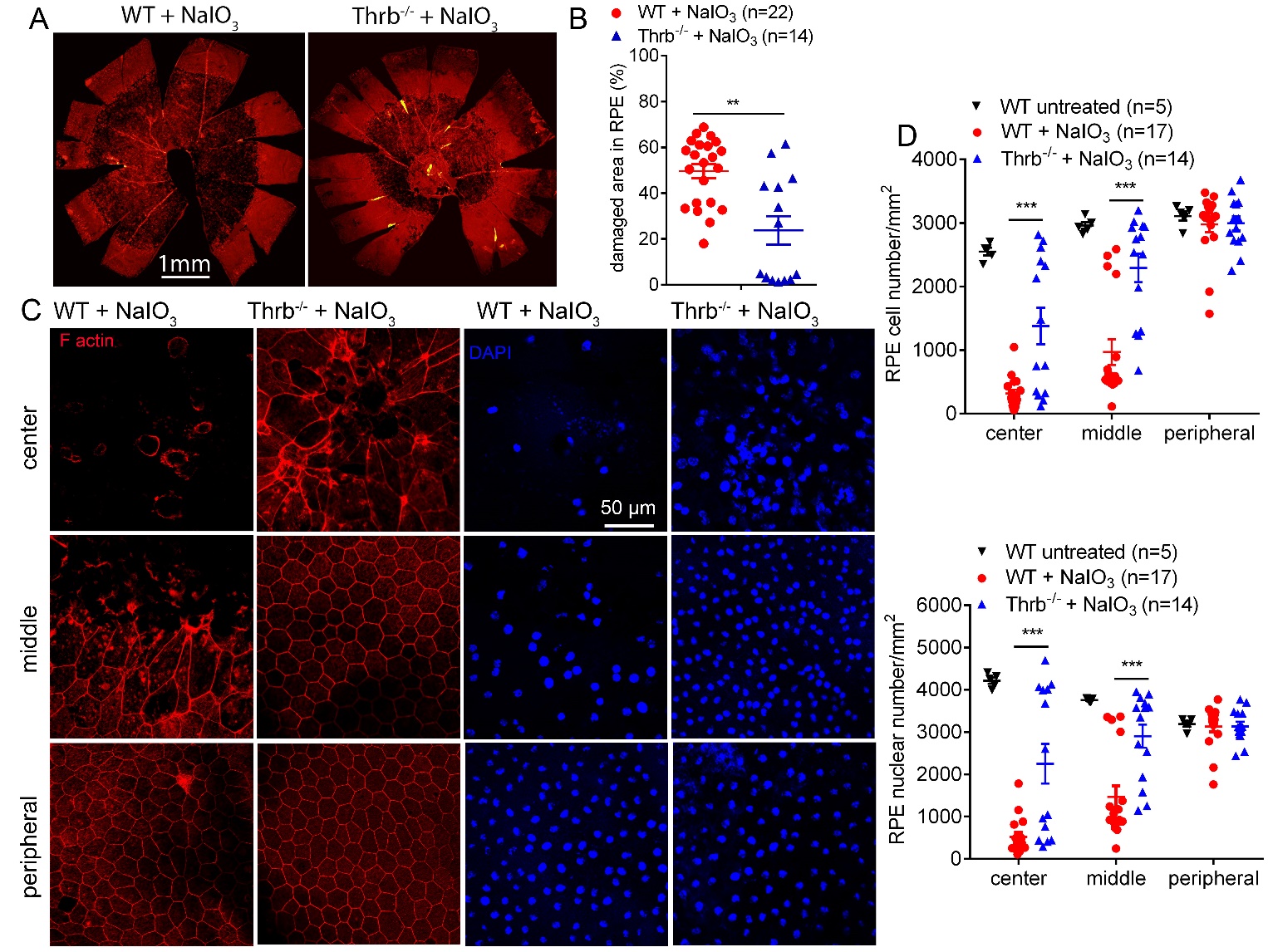


Figure 4


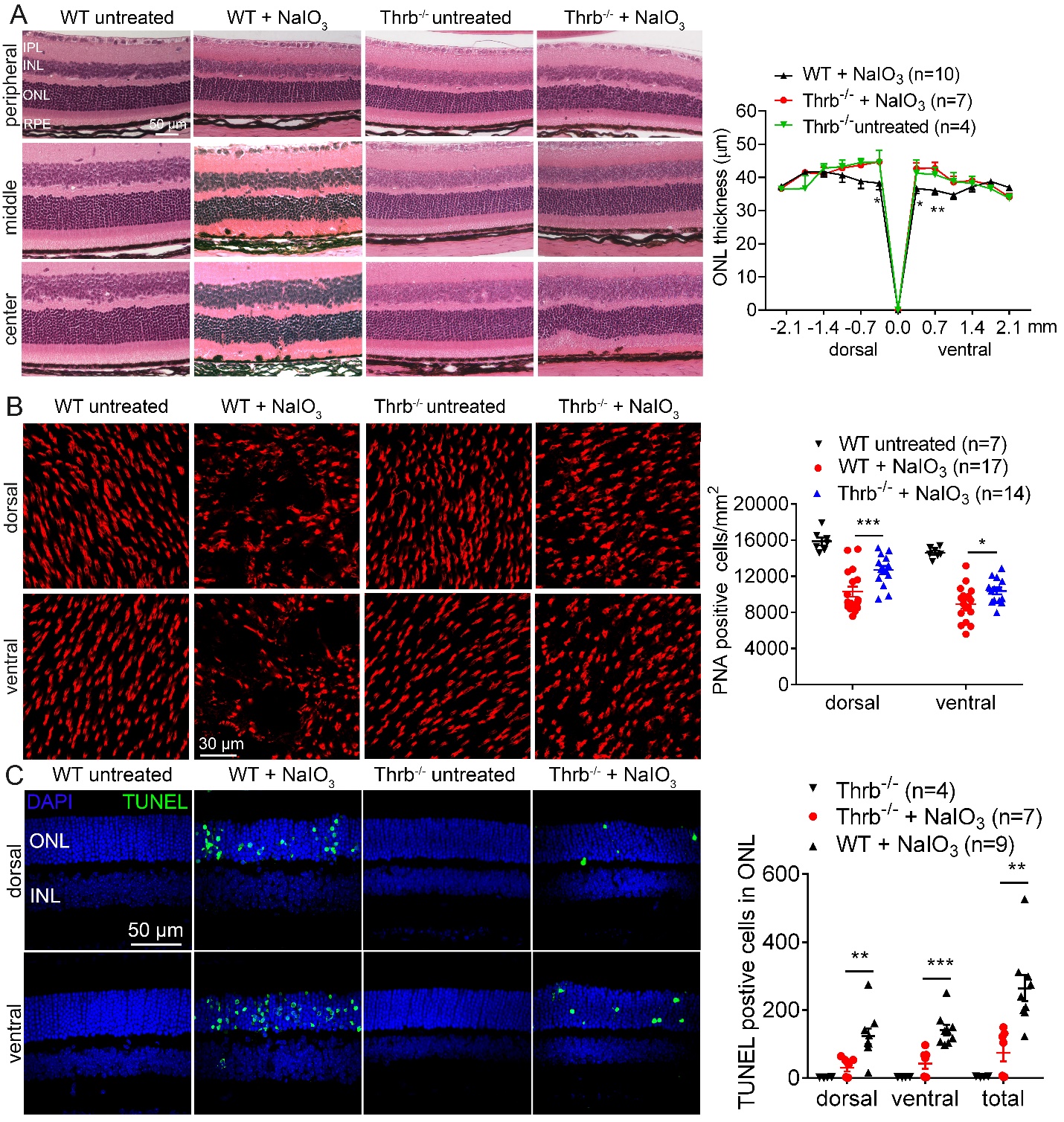


Figure 5


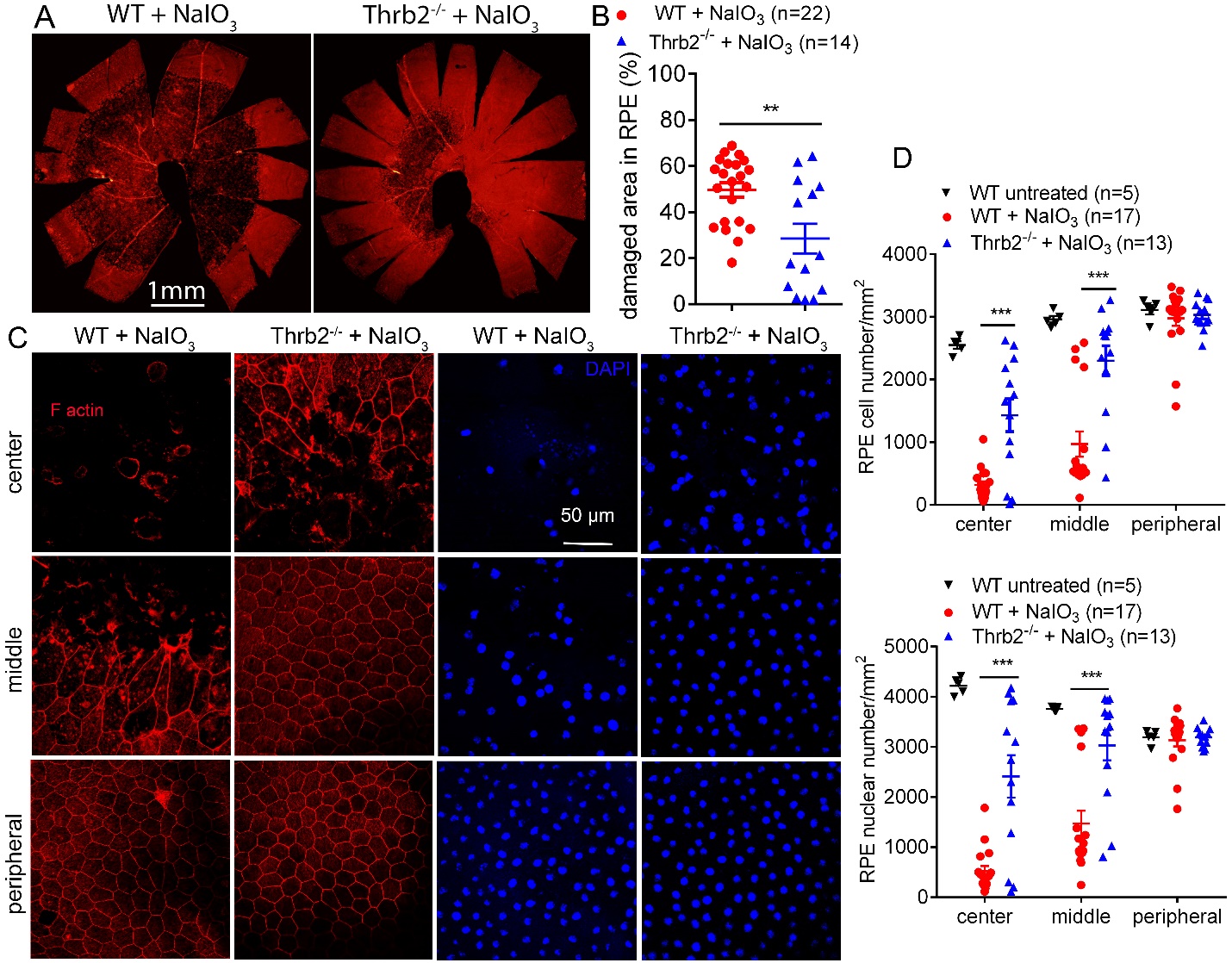


Figure 6


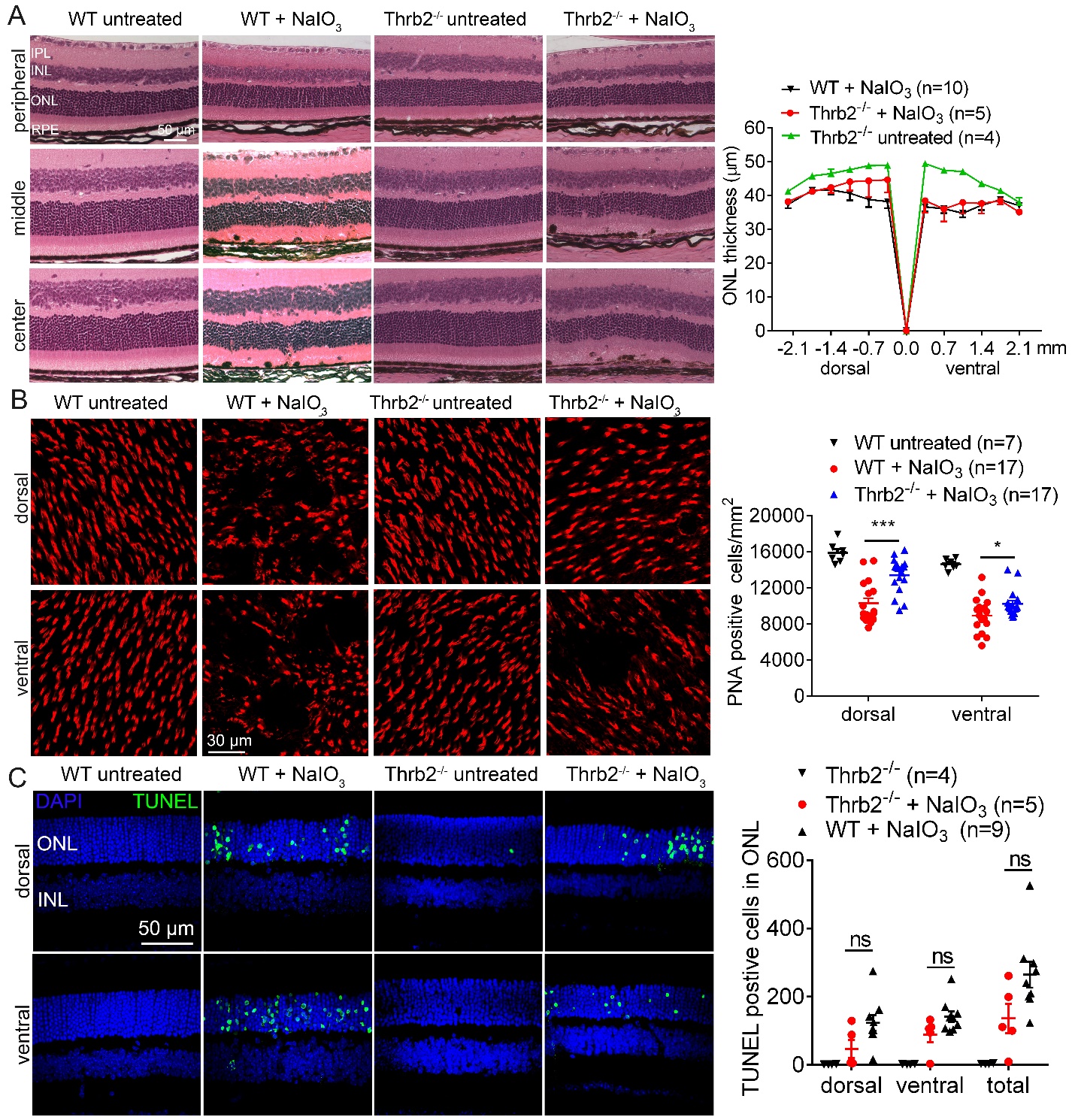


Figure 7





Figure 8
